# The *G123* rice mutant, carrying a mutation in *SE13*, presents alterations in the expression patterns of photosynthetic and major flowering regulatory genes

**DOI:** 10.1371/journal.pone.0233120

**Published:** 2020-05-18

**Authors:** Juan Luis Reig-Valiente, Carles Borredá, Manuel Talón, Concha Domingo

**Affiliations:** Centro de Genómica, Instituto Valenciano de Investigaciones Agrarias, Moncada, Spain; Kyung Hee Univeristy, REPUBLIC OF KOREA

## Abstract

Day length is a determinant of flowering time in rice. Phytochromes participate in flowering regulation by measuring the number of daylight hours to which the plant is exposed. Here we describe *G123*, a rice mutant generated by irradiation, which displays insensitivity to the photoperiod and early flowering under both long day and short day conditions. To detect the mutation responsible for the early flowering phenotype exhibited by *G123*, we generated an F_2_ population, derived from crossing with the wild-type, and used a pipeline to detect genomic structural variation, initially developed for human genomes. We detected a deletion in the *G123* genome that affects the *PHOTOPERIOD SENSITIVITY13* (*SE13*) gene, which encodes a phytochromobilin synthase, an enzyme implicated in phytochrome chromophore biosynthesis. The transcriptomic analysis, performed by RNA-seq, in the *G123* plants indicated an alteration in photosynthesis and other processes related to response to light. The expression patterns of the main flowering regulatory genes, such as *Ghd7*, *Ghd8* and *PRR37*, were altered in the plants grown under both long day and short day conditions. These findings indicate that phytochromes are also involved in the regulation of these genes under short day conditions, and extend the role of phytochromes in flowering regulation in rice.

## Introduction

An optimal flowering time, or heading date, that adjusts to local agroclimatic conditions is essential for maximizing the yield potential of rice crops. In line with this, flowering regulation in rice has played an important role in its expansion and diversification, and is one of the main factors that contributes to the adaption of this crop in northern regions [1]. Rice domestication took place in a region with a tropical climate with a short day (SD) length and temperatures that only slightly vary all year long [2]. During its expansion, rice crops reached northern areas where permissive temperatures occur only in summer, when day length is long. In these regions, rice plants have adapted to long days (LD) in summer, to mainly avoid cold winter temperatures, via an artificial selection process to modify flowering regulation. This adaptation process consisted in chiefly avoiding flowering inhibition under LD conditions and sensitivity to the photoperiod being gradually lost [1].

The transition from the vegetative to the reproductive phase in rice is governed by the action of two master genes, *Heading date 3a* (*Hd3a*) and *RICE FLOWERING LOCUS T 1* (*RFT1*), which encode the florigens, mobile molecules that signal flowering. A fine-tuned complex regulation of the expression of both these genes governs flowering in rice plants, with the main inducers being *Hd3a* under SD conditions and *RFT1* under LD conditions [3]. The expressions of *Hd3a* and *RFT1* are mainly modulated by two genes that represent two independent regulatory pathways: *Early heading date 1* (*Ehd1*), which acts as an integrator of different signals; *Heading date 1* (*Hd1*), regulated by the circadian cycle. Although the *Hd1* and *Ehd1* regulatory pathways were initially considered independent, recent data indicate that *Hd1* is able to regulate *Ehd1* expression levels [4]. *Ehd1* induces flowering and its expression is regulated by several factors. In contrast, *Hd1* is a flowering inhibitor under LD conditions that it is regulated by the circadian clock through *OsGIGANTEA* (*OsGI*) [5]. The existence of several non-functional alleles in cultivars grown at northern latitudes has led other authors to propose that the lack of functional Hd1 to be one of the mechanisms of adaptation to regions with LD characteristics of a temperate climate [6]. Other studies have proposed a model in which flowering is regulated by the action of individual genes on *Ehd1*, which acts as a central node that integrates floral repressive signals in the absence of a functional Hd1 [7]. In fact, some cultivars grown at northern latitudes carry a functional *Hd1* allele and are still able to flower under LD conditions [6]. Another actor in flowering regulation is *Ghd7* (*Grain number plant height and heading date 7*), a flowering inhibitor that it is expressed mainly under LD conditions and negatively modulates *Ehd1* expression and, consequently, hinders *Hd3a* induction [8].

Recent studies have revealed that some major regulatory proteins modulate floral transition by forming different activation or repression complexes [9]. This is the case of Hd1, which can activate or inhibit flowering depending on day length. Hd1 forms heterodimeric complexes with Ghd7, a protein containing a CCT domain, which interacts with the *Ehd1* promoter by repressing its expression in the morning under LD conditions [10]. In the absence of a functional *Ehd1* and under LD conditions, Hd1 acts as a strong repressor [11; 12], while the activating function occurs at night and independently of day length conditions. *Ghd7* is able to repress *Ehd1*, *Hd3a* and *RFT1* alone in the morning independently of day length. This fact suggests that there are other proteins which interact with Ghd7 to perform this function. The expressions of *Hd1* and *Ghd7* are independently regulated. However, the repressor activity of the Hd1-Ghd7 complex can be modulated through the action of Phytochrome B (PhyB) [13].

Phytochromes are responsible for red and far-red light perception, and play an important role in photoperiodic flowering regulation in rice [14]. Three phytochromes have been described in rice: PhyA, PhyB and PhyC. A mutation in either *PhyB* or *PhyC* causes moderate early flowering under LD conditions, while a mutation in *PhyA* barely has any effect on flowering time, which indicates that the presence of PhyB and PhyC is essential for inhibiting flowering in the LD photoperiod [15]. Furthermore, the rice phytochrome triple mutant (*phyAphyBphyC*), which completely lacks any phytochrome, exhibits very early flowering [14]. Phytochromes play a supporting role in flowering regulation in plants. In Arabidopsis, the stability of CONSTANS (CO), the homolog of Hd1 in rice, is a key feature in flowering regulation mediated by the photoperiod [16]. The CO protein is unstable at night and in the morning, when it is quickly degraded, but remains stable in the afternoon. Consequently, CO is abundant in the light phase under LD [16]. It has also been demonstrated that PhyB physically interacts with E3 ubiquitin ligase HIGH EXPRESSION OF OSMOTICALLY RESPONSIVE GENES1 (HOS1) and CO to form a three-protein complex that coordinates the photoperiodic response [17]. In rice, phytochromes inhibit flowering by negatively modulating both the *Hd1* and *Ehd1* flowering pathways. Furthermore, PhyA homodimers and PhyB-PhyC heterodimers are independently sufficient to activate *Ghd7* transcription, while PhyB homodimers can repress it [13]. More recently, PhyA, PhyB and OsGI, a circadian oscillator protein, have been described to interact with Ghd7 [18].

There is direct evidence that phytochromes control the flowering signaling pathway through *PHOTOPERIOD SENSITIVITY 5* (*SE5*) by encoding a heme oxygenase that converts the heme group into biliverdin IX α during phytochromobilin (PƟB) biosynthesis, a phytochrome chromophore [19]. It has been reported that *SE5* negatively controls *Ehd1* expression and, thus, inhibits flowering. Furthermore, *SE5* confers photoperiodic sensitivity through the regulation of *Hd1* [20]. Mutants defective in *SE5* are deficient in active phytochromes and exhibit very early heading under both SD and LD conditions. Furthermore, the deficiency of both PhyA and PhyB in *se5* plants results in a light response being absent in the mutant [19]. Similarly, *PHOTOPERIOD SENSITIVITY 13* (*SE13/OsHY2*) encodes a PƟB synthase that participates in the final step of PƟB synthesis [21]. Phytochrome-defective plants, as a consequence of lack of the functionality of *SE13/OsHY2*, flower earlier than those plants with functional phytochromes [21], which occurs in *se5* mutants.

In order to understand the factors involved in photoperiodic flowering regulation in rice, we characterized *G123*, an early flowering mutant that derives from the Gleva variety that is widely cultivated in Spain. We determined the changes in the expression patterns of the main flowering regulatory genes in this mutant, and carried out a comparative transcriptomic analysis with the parental variety. The genome structure analysis allowed us to identify the mutation responsible for the early heading phenotype displayed by this mutant.

## Materials and methods

### Plant material and growing conditions

Seeds of the Gleva variety were irradiated with fast neutrons 25 Gy at the Instituto Tecnologico e Nuclear (Sacavem, Portugal), and were germinated and grown in pots in a greenhouse at a controlled temperature (25°C) and relative humidity (50% RH) under natural daylight conditions (latitude 39° 28’ N). Adult plants were grouped into families of five plants and their seeds were collected. One hundred and twenty-two M_2_ plants from each family were grown in rows, spaced 20 x 20 cm, in the field. They were screened for those plants showing earlier flowering than Gleva. The M_3_ plants were cultivated in summer in pools resembling cultivation field conditions and flowering dates were recorded. Other traits, such as height, panicle length, number of panicles and grain weight per plant, were also scored.

For the photoperiod sensitivity assays, the gene expression analysis, RNA-seq and mutation detection, plants were cultivated in growth chambers (SANYO Mod. MLR350) equipped with broad-spectrum fluorescent tubes (400–700 nm) (GROLUX F36W / GRO-T8, Sylvania, Germany) with a light intensity of 250 μmol^-2^ · s^-1^. Plants were cultivated separately for each analysis. Plants were grown under LD (14 h light:10 h dark) or SD (10 h light:14 h dark) conditions, or under 12 h light:12 h dark photoperiod conditions. Temperature was kept constant at 27°C in all the experiments. To monitor the photoperiod effect on minimizing the differences in the flowering induction times, the seeds of Gleva and *G123* were sown in pots and grown under 12 h light:12 h dark photoperiod conditions for 4 weeks, followed by 1 week under the LD or SD conditions. For the expression pattern analysis, at the end of week 5, the time series of the samples were taken from the second leaf of three different plants every 4 h. The time when plants began to receive light was considered 0 h. For the RNA-seq analysis, a new set of plants was grown and the second leaves of these plants were collected 20 h after dawn. Samples were frozen in liquid nitrogen and stored at -80°C until the RNA extraction procedure.

Three hundred sixty-five F_2_ plants derived from a cross between Gleva and *G123* were grown in pots in a greenhouse under natural light conditions in summer. The heading date was considered the time when half of the first panicle emerged. The plants that flowered before 72 DAS sowing were considered the early flowering plants. A chi-square test was used to test the hypothesis of a single recessive gene.

### RNA isolation

For the quantitative Real-Time PCR, total RNA was isolated using extraction buffer (0.1 M LiCl; 0.1 M Tris pH8; 1% SDS; 0.01 M EDTA) and a mixture of phenol: chloroform: isoamyl alcohol (25: 24: 1), and was then precipitated with LiCl at a final concentration of 2M LiCl and resuspended in TE. The RNA concentration was measured using the QubitTM RNA BT Assay Kit (Ref: Q10211) following the manufacturer's instructions, and was measured by Qubit® 2.0 Fluorometer (Life Technologies, USA).

The RNA isolation for the RNA-seq analysis was performed using the NucleSpin® RNA plant Kit (Ref: 740949.50, MACHEREY-NAGEL, Germany) following the manufacturer's instructions. The quality and concentration of RNA were tested by agarose gel electrophoresis with a BioAnalyzer 2100 (Agilent) and a NanoDrop ® spectrophotometer (Thermo Scientific). mRNA was enriched using oligo-dT beads.

### Quantitative real-time PCR

The gene expression analyses were carried out on 2 replicates using RNA extracted from the second leaf of 2 different plants. The analyses were performed in a single step with the Light Cycler® Fast Start DNA MasterPlus SYBR Green I Kit (Applied Biosystems TM, Ref: 03515885001) following the manufacturer’s instructions. First-strand cDNA was synthesized from 100 ng of total RNA by reverse M-MuLV Roche® transcriptase. The Real-Time PCR procedure involved incubation at 48°C for 30 min and 95°C for 10 min, followed by 45 cycles at 95°C for 2 s, 55–61°C for 8 s and, finally, 72°C for 8 s. Next samples were incubated at 95°C for 15 seconds and 42°C for 1 min, followed by a temperature gradient from 42°C to 95°C with a ramp of 0.1°C/s. Fluorescence intensity was measured during both the extension at 72° C and the temperature gradient. The specificity of the reaction was verified by a melting curve analysis, obtained during the temperature gradient and by sequencing the reaction product. The expression of a rice ubiquitin gene was used for normalization purposes. The sequences of primers, extension times and number of cycles are provided in S1 Table.

### mRNA-Seq library construction and sequencing

mRNA-Seq library construction and sequencing were performed by Novogen Bioinformatics Technology Co., Ltd (Hong Kong). Briefly, a library of insert size 250 ~ 300 bp was constructed, followed by its sequencing by pair-end readings of 150 bp. Following random mRNA fragmentation, cDNA was synthesized by using random hexamer primers and reverse transcriptase. Then the synthesis of the complementary strand was carried out following the Illumina mRNA Sequencing Sample Preparation Guide. A series of the end terminal repair and ligation of the pair-end sequencing adaptors was performed. The employed adapters were: 5' adapter 5'-AATGATACGGCGACCACCGAGATCTACACTCTTTCCCTACACGACGCTCTTCCGATCT and 3' adapter 5'-GATCGGAAGAGCACACGTCTGAACTCCAGTCACATCACGATCTCGTATGCCGTCTTCTGCTTG, where the six underlined bases correspond to the index. Size selection and PCR amplification enrichment were performed. The quality testing of the library was done using Qubit 2.0 and Bioanalyzer 2100, and the effective concentration of the library was quantified accurately by Q-PCR.

The mRNA-Seq libraries were sequenced by an Illumina HiSeq/MiSeq sequencer. Raw readings were filtered to eliminate low quality readings and adapters. Those readings containing sequences of adapters, or 10% of their indeterminate bases or more than 50% of low quality bases (Qscore < = 5), were eliminated.

### RNA-Seq and differential expression analysis

Prior to the differential expression analysis, an extra quality analysis was performed using the FastQC High Throughput Sequence QC Report (version: 0.11.5, www.bioinformatics.babraham.ac.uk/projects/). Version 7.5.2 of the CLC Genomics Workbench software (QIAGEN, Germany) [22] was used for the differential expression analysis. Reads were filtered according to default parameters for Illumina reads, plus a restriction of a 250–300 bp distance between pairs. Reads were cut based on their quality with a limit of 0.049 and a maximum number of ambiguous nucleotides that equaled 2. In addition, 15 nucleotides of the 5’ end of all the reads were first trimmed due to discrepancy in the percentage of bases according to the FastQC reports. The release 7th of the rice pseudomolecules and genome annotation data was used as a reference. The mapping and distribution of reads across genes were carried out with default parameters. Expression levels were normalized by RPKM (*Reads Per Kilobase per Million mapped reads*). The differential expression analysis was performed by employing a re-implementation of the “Exact test” of the edgeR Bioconductor package [23] for a two-group comparison with a *common dispersion cutoff* of 5 and p-values with FDR correction. The significance threshold was set at FDR < 0.1.

Functional annotation according to Gene Ontology (GO), and the enrichment analysis, were carried out through the CARMO platform (Comprehensive Annotation of Rice Multi-Omics data) [24] (http://bioinfo.sibs.ac.cn/carmo/). The GO terms with FDR < 0.05 were considered enriched.

### Nuclear genome DNA extraction and genome sequencing

Nuclear genome DNA was isolated from 2 g of fresh leaves from Gleva, *G123* and from the 20 F_2_ plants showing an early phenotype by following a modified CTAB protocol [25]. A nuclear DNA mixture (Epool), in equal amounts, was prepared from the 20 F_2_ individuals that presented the same phenotype as the mutant.

The final concentration and quality of DNA were checked with the Qubit™ dsDNA BR Assay Kit (Ref: Q32853) following the manufacturer’s instructions and using Qubit^®^ 2.0 Fluorometer (Life technologies, USA).

Library construction and genome sequencing were carried out at Novogen Bioinformatics Technology Co., Ltd as follows: the DNA from each sample was cut into fragments of approximately 350 bp, which were used to construct a genomic DNA library using the NEBNext® DNA Library Prep Kit following the manufacturer's instructions. In the next step, the repair of ends, addition of dAMP tails (dA-tailing), and further ligation with the NEBNext adapter were done, and the required fragments (300–500 bp) were enriched by the P5 and P7 indexed oligos. After purification and quality checking, the resulting library was ready for sequencing.

The quality control of the library was first performed with a Fluorometer Qubit®2.0 and Agilent® 2100 bioanalyzer. Finally, the real-time PCR (qPCR) was performed to detect the effective concentration of each library. The libraries with an appropriate insert size (~ 350 bp) and effective concentration above 2 nM were selected and mixed according to their effective concentration and the expected amount of data to be produced. The sequencing of the pair-end readings was performed on the Illumina®sequencing platform with a reading size of PE150pb at each end. The raw data obtained from sequencing were filtered to discard the paired readings showing contamination with adapters, or if indeterminate nucleotides constituted more than 10% of the sequence, or if nucleotides with a low quality (quality of the bases less than 5, Q <5) constituted more than 50% of the reading. DNA sequencing generated 44.7 Gb of clean reads. The sequencing and cleaning process statistics are summarized in S2 Table.

### Detection of the mutation

Raw sequencing data were filtered by applying a minimum sequencing quality threshold of 30 in at least 70% of the read length. Those reads that did not fulfill these conditions, as well as their pairs, were discarded. Then reads were mapped using bwa mem with an increased reseeding (-r 1.2) against the reference genome Os-Nipponbare-Reference-IRGSP-1.0 v7 (http://rice.plantbiology.msu.edu/). The resulting BAM files were processed using samtools [26]. The mapping statistics are summarized in S3 Table.

Structural variations were detected using LUMPY [27] in the multisample mode with the default parameters by analyzing together the Gleva, *G123* and Epool genomes. The resulting VCF was genotyped by SVTYPER [28]. This generated a VCF, including the putative SVs found in any of the three genomes compared to the employed reference genome.

Finally, SVs were filtered using the allele balance (AB) reported by SVTYPER; that is, the proportion of reads supporting the variation against the total reads for each sample. The SVs with an AB<0.001 (virtually no read supported variation) for Gleva and an AB>0.99 for *G123* and Epool were selected by SnpSift [29]. The selected variations, which were absent in Gleva, but present in *G123* and Epool, were manually verified with the genome visor IGV Browser to check for false-positives. The data from the statistics analysis of the detected SV are summarized in S4 Table.

RNAseq and genomic sequencing data for the mutation identification have been deposited at the European Nucleotide Archive (ENA) in the European Bioinformatics Institute (EBI) with the accession number PRJEB37950.

## Results

### Characterization of *G123*, a rice mutant displaying early flowering and insensitivity to the photoperiod

The *G123* mutant was isolated as an early flowering mutant in the field screenings, under natural LD conditions, from a mutant M_2_ population that derived from the irradiation of Gleva, a local *temperate japonica* cultivar widely grown in Spain. The *G123* plants flowered in the field 82 days after sowing (DAS), which was 1 week earlier than the wild-type plants.

**Fig 1:**
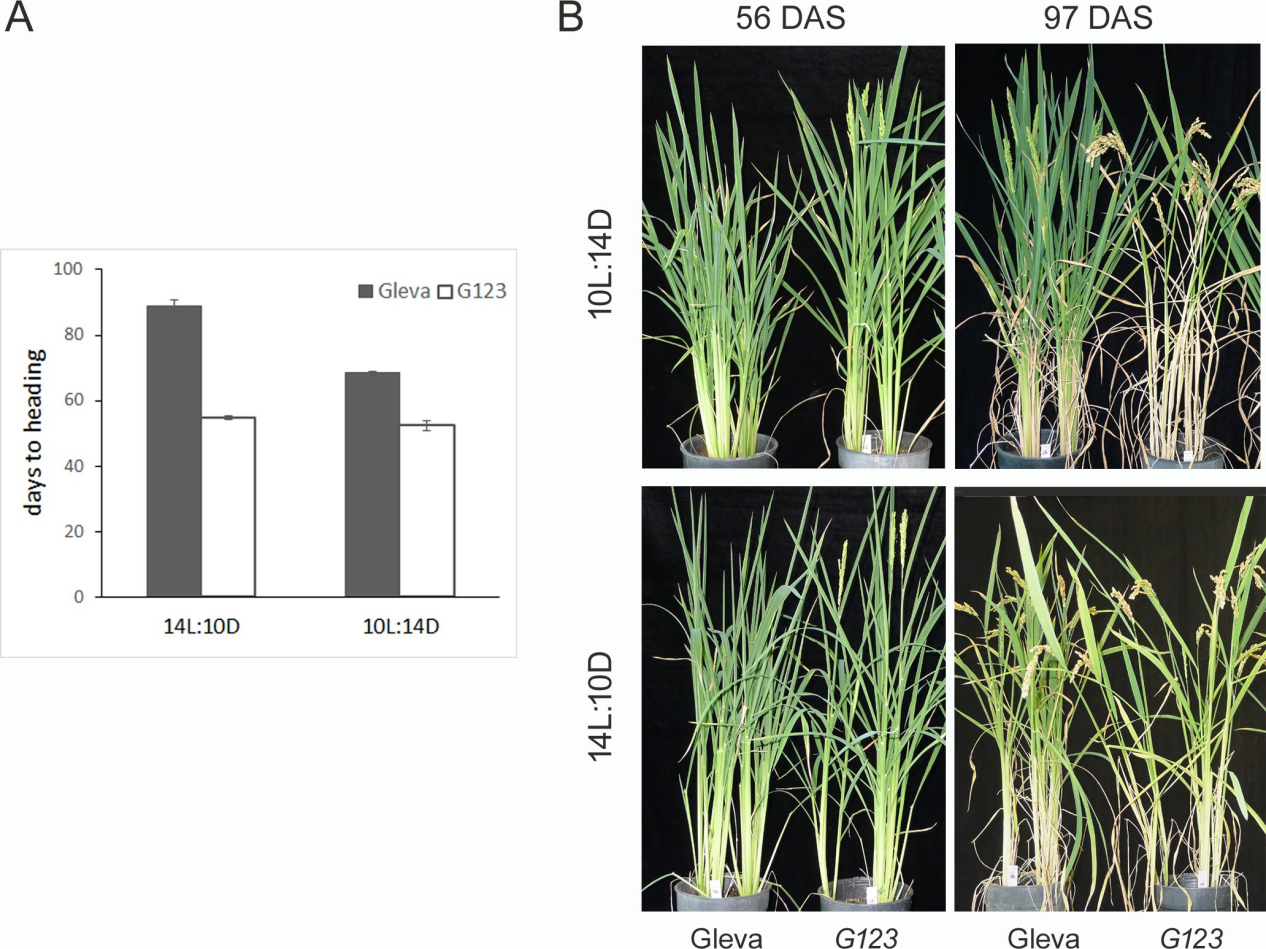
Comparison of the flowering phenotype of the *G123* mutant and Gleva wild-type plants. (A) Days to heading under SD (10L:14D) or LD (14D:10D) photoperiod conditions. Days to heading were scored from germination to emergence of panicle from the main culm. (B) Phenotypes of the Gleva wild type and *G123*. Plants were grown for 4 weeks under 12 h light:12 h dark photoperiod and then transferred to SD or LD conditions. Plants at 56 or 97 days after sown are shown.

Exposure to different photoperiod conditions showed that the *G123* plants were insensitive to the photoperiod because the number of hours of light did not affect the heading date. Plants were cultivated in growth chambers under LD (14 h light:10 h dark) and SD (10 h light:14 h dark) conditions, and the heading date was recorded. Under both SD and LD conditions, the *G123* plants flowered 53 days after germination, while the Gleva plants flowered 68 days under SD conditions and 89 days after germination under LD conditions (Fig 1A). The *G123* plants were consistently shorter and displayed a slightly yellowish color compared to the wild-type plants. They also developed more panicles, but the total grain weight lowered by 25.1% (Fig 1B and Table 1).

**Table 1:** Phenotypic characteristics of the Gleva and *G123* plants. Height, panicle length, number of panicles and grain weight per plant are indicated.

	Height (cm)	Panicle length (cm)	Number of panicles	Grain weight (g)
***G123***	66.1±5.8	13.0±0.4	20.5±3.5	47.4±9.2
**Gleva**	67.9±3.7	13.1±0.5	15.8±2.6	63.3±7.6

**Fig 2:**
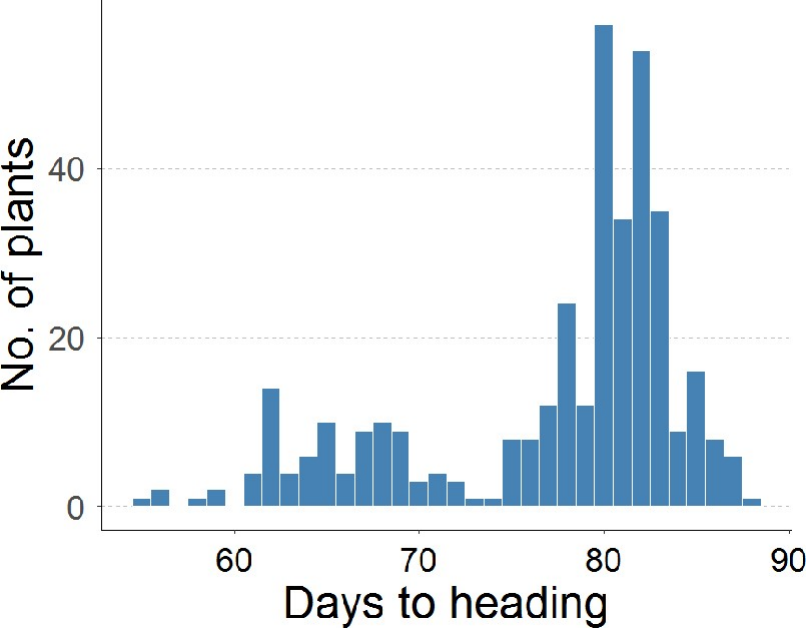
Distribution of days to heading of an F_2_ population derived from a cross between Gleva and *G123*.

To determine whether the observed early flowering phenotype was due to a recessive mutation in a single gene, the 365 F_2_ plants derived from a cross between *G123* and Gleva were grown in pots in a greenhouse under natural light conditions in summer, and the heading date was recorded. As seen in Fig 2, the flowering frequencies showed a bimodal distribution. The progeny segregated a 289:82 ratio for the plants with a vegetative cycle that was longer or shorter than 72 DAS, respectively, with a 3:1 segregation ratio (chi-squared test: χ^2^ = 1. 8, p = 0.18). This indicated that the flowering early phenotype in *G123* is conferred by a single recessive mutation.

### The expression of the flowering regulatory genes is altered in the *G123* mutant

**Fig 3:**
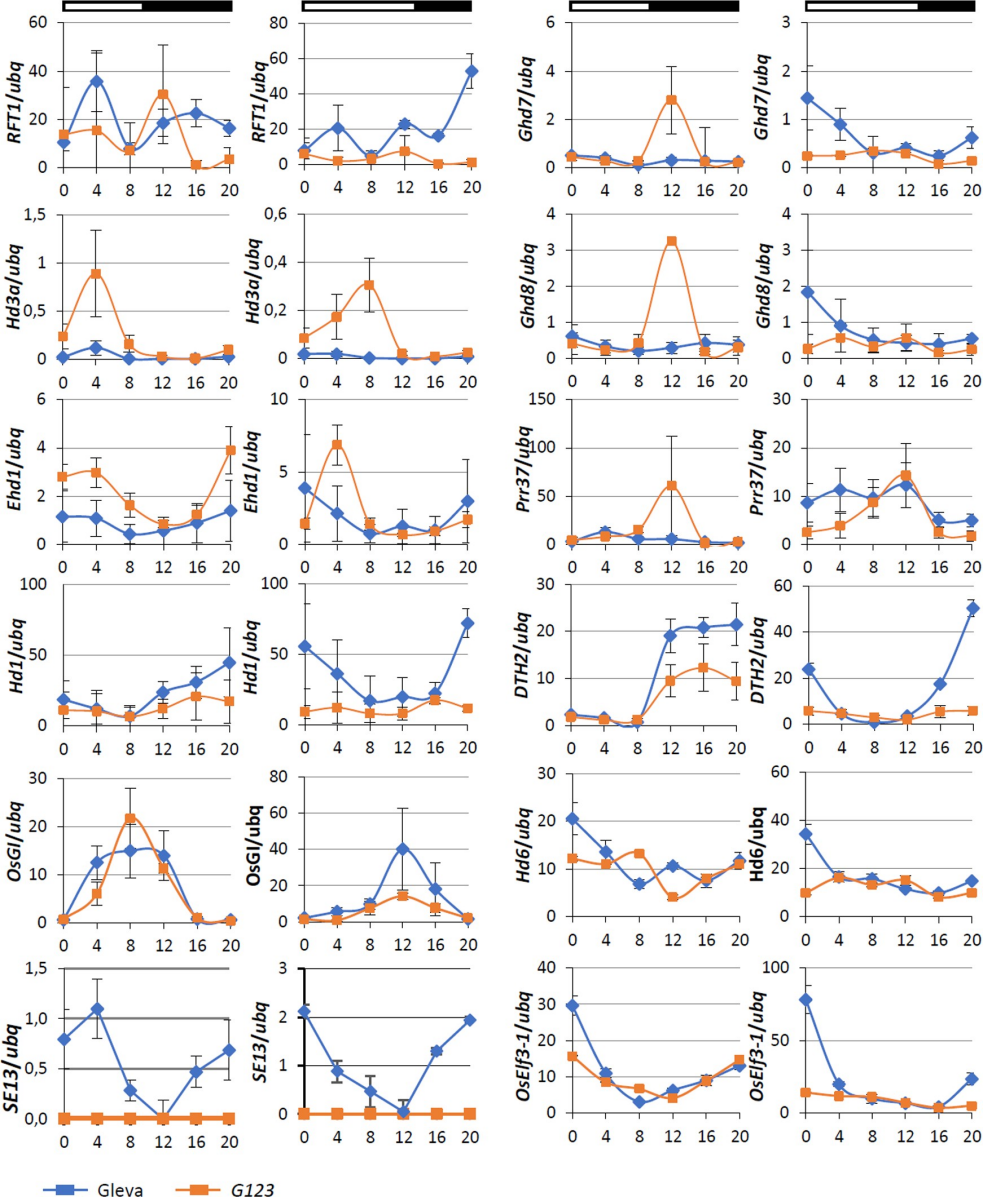
Diurnal expression patterns of flowering regulatory genes in Gleva and *G123*. Plants were grown under 12 h light:12 h dark day conditions for 4 weeks, followed by an additional 1-week period under LD or SD conditions. White and black horizontal bars represent the light and dark periods, respectively. Each value is the average of three biological replicates. Error bars indicate standard deviation from the mean.

The daily expression pattern of the main genes involved in the photoperiod regulation of flowering in the Gleva and *G123* plants reflected their flowering phenotype differences (Fig 3). *Hd3a* showed higher expression levels in *G123* than in Gleva at 4 h under SD and at 8 h under LD conditions after dawn. The *RFT1* expression levels in the *G123* plants grown under the SD conditions were lower than in Gleva at the end of the dark period, but under LD conditions, the *RFT1* levels were generally lower throughout the photoperiod. Under SD conditions, *Ehd1* expression was generally higher in *G123* than in Gleva, and increased in the dark phase and decreased in the light phase. Under LD conditions, the levels of *Ehd1* mRNA were similar in both varieties, except at 4 h after dawn when *Ehd1* expression peaked for *G123*. Both lines exhibited similar *Hd1* expression pattern under both LD and SD conditions, except at the end of the day, when the *G123* plants showed lower levels than the wild type. We also analyzed the expression of the other genes involved in flowering regulation that modulated *Hd1* and *Ehd1* expressions to some extent (Fig 3). *OsGI* displayed similar expression patterns in *G123* and Gleva under both SD and LD conditions, with expression peaks at 8 h and 12 h, respectively. *Ghd7* and *Ghd8*, both inhibitors of flowering under LD conditions, generally exhibited similar and rather constant expression profiles in both varieties. However, under the SD conditions, both genes showed a peaked expression in *G123* in the dark phase 12 h after dawn. Under LD conditions, the *Ghd7* and *Ghd8* expression levels at dawn tended to be lower in *G123* than in Gleva. This expression pattern was also observed in the *Pseudo-Response Regulators 37* (*OsPRR37*) coding for a protein with a CCT domain, whose expression is governed by the circadian clock [30]. The expression levels of *DTH2* in both *G123* and Gleva increased in the dark under SD conditions, but remained low in the mutant under LD conditions. The *OsEARLY FLOWERING3-1* (*OsELF3-1*) expression was also activated in the dark in both *G123* and Gleva under SD, and levels lowered in the daytime, but the expression remained low in *G123* under the LD conditions. *Hd6* expression remained low in the *G123* plants, but exhibited a peak at 0 h in the Gleva plants under both SD and LD conditions (Fig 3). *SE13* expression was also analyzed and increasing expression levels were observed at night, which lowered in the daytime under both SD and LD conditions in Gleva plants. No expression was observed in the *G123* plants (Fig 3).

### RNA-seq transcriptome analysis

To study the effect caused by the mutation on the transcriptome, an RNA-seq experiment was performed to detect the genes differentially expressed between *G123* and Gleva. *G123* and Gleva were exposed during one week to LD conditions, and mRNA was isolated from leaf samples 20 h after dawn, when the expression of the two pivotal regulatory genes, *RFT1* and *Hd3a*, in Gleva and *G123* was clearly different. An RNA-seq analysis of the differential gene expression was performed. A threshold of 1.5-fold and an FDR <0.1 were set to evaluate the differential gene expression. Following this criterion, the analysis revealed that 116 genes were differentially expressed between both genotypes, of which 62 were up-regulated and 54 were down-regulated in *G123 versus* Gleva (Table 2).

**Table 2:** List of the differential expressed genes between Gleva and *G123*. The tagwise dispersions using the EDGE test for fold change, p-value and FDR p-value correction are shown. Genes included in different classification groups are indicated as: (f) flowering, (t) transport, (p) photosynthesis, light harvesting, and responses to blue, red and far red light, (m) membrane, (pl) plastid and chloroplast and (c) cytoplasm components.

Feature ID	Gene annotation	Fold change	P-value	FDR p-value correction	
LOC_Os04g58200	protochlorophyllide reductase A, chloroplast precursor, putative	69.41	4.10E-09	0.000	p, m, pl
LOC_Os06g18670	anthocyanidin 3-O-glucosyltransferase, putative	40.48	3.74E-05	0.027	
LOC_Os03g28170	expressed protein	38.95	2.48E-06	0.003	
LOC_Os09g25810	nodulin, putative	38.12	8.26E-06	0.008	m
LOC_Os06g06320	Hd3a/OsFTL2 FT-Like2 homologous to the Flowering Locus T gene; contains Pfam profile PF01161: Phosphatidylethanolamine-binding protein	37.86	3.67E-08	0.000	f
LOC_Os07g34570	FAD-dependent oxidoreductase domain containing protein	33.38	5.67E-19	0.000	t, m, pl
LOC_Os03g39610	chlorophyll A-B-binding protein, putative	27.57	4.29E-22	0.000	p, m, pl
LOC_Os09g17740	chlorophyll A-B-binding protein, putative	23.44	7.77E-15	0.000	p, m, pl
LOC_Os03g54160	OsMADS14—MADS-box family gene with MIKCc type-box	20.53	1.58E-06	0.002	f
LOC_Os05g06780	LTPL104—Protease inhibitor/seed storage/LTP family protein precursor	20.32	5.77E-05	0.039	t
LOC_Os04g38220	transporter family protein, putative	16.90	9.39E-06	0.009	t, m
LOC_Os01g41710	chlorophyll A-B binding protein, putative	14.83	4.89E-10	0.000	p, m, pl
LOC_Os02g45225	expressed protein	12.51	3.17E-05	0.024	
LOC_Os05g38680	plant-specific domain TIGR01589 family protein	12.47	3.11E-06	0.004	
LOC_Os03g06570	IQ calmodulin-binding motif family protein, putative	10.18	1.70E-04	0.086	
LOC_Os01g52250	starch synthase, putative	9.29	1.06E-06	0.002	pl
LOC_Os08g33820	chlorophyll A-B binding protein, putative	9.28	6.82E-07	0.001	p, m, pl
LOC_Os05g51150	RNA polymerase sigma factor, putative	8.52	9.00E-07	0.001	p. pl
LOC_Os03g28160	jacalin-like lectin domain containing protein	7.91	2.40E-05	0.019	
LOC_Os04g32850	basic proline-rich protein, putative	7.71	6.11E-07	0.001	t
LOC_Os06g01250	cytochrome P450, putative	7.24	4.08E-06	0.004	
LOC_Os04g52260	LTPL124—Protease inhibitor/seed storage/LTP family protein precursor	7.01	2.18E-07	0.000	t
LOC_Os07g37550	chlorophyll A-B binding protein, putative	6.55	2.13E-06	0.003	p, m, pl
LOC_Os01g08020	boron transporter protein, putative	6.54	2.01E-04	0.098	t, m
LOC_Os04g08828	cytochrome P450, putative	6.43	1.07E-06	0.002	pl
LOC_Os03g29770	EF hand family protein	6.35	2.64E-05	0.020	
LOC_Os11g13890	chlorophyll A-B-binding protein, putative	5.76	2.07E-10	0.000	p, m, pl
LOC_Os01g60730	RING-H2 finger protein, putative	5.39	8.82E-06	0.008	
LOC_Os04g57880	Heat-shock protein DnaJ, putative	4.79	1.67E-07	0.000	pl
LOC_Os06g21590	chlorophyll A-B-binding protein, putative	4.71	4.18E-05	0.030	p, m, pl
LOC_Os11g31190	nodulin MtN3 family protein, putative	4.54	6.42E-07	0.001	t, m
LOC_Os12g37510	UDP-glucoronosyl and UDP-glucosyl transferase domain-containing protein	4.53	1.23E-04	0.069	
LOC_Os01g37590	peptide transporter PTR2, putative	4.52	9.44E-07	0.001	t, m
LOC_Os01g63190	laccase precursor protein, putative	4.45	6.22E-06	0.006	
LOC_Os06g06290	GDSL-like lipase/acylhydrolase, putative	4.38	1.32E-12	0.000	
LOC_Os05g28740	universal stress protein domain-containing protein, putative	3.98	3.33E-05	0.025	
LOC_Os02g15120	RING-H2 finger protein, putative	3.92	6.50E-06	0.007	
LOC_Os05g02070	expressed protein	3.91	8.62E-10	0.000	
LOC_Os02g10390	chlorophyll A-B-binding protein, putative	3.85	1.90E-08	0.000	p, m, pl
LOC_Os07g41370	OsMADS18—MADS-box family gene with MIKCc type-box	3.82	2.48E-08	0.000	f
LOC_Os03g08470	AP2 domain-containing protein	3.81	3.33E-07	0.001	pl
LOC_Os03g06520	sulfate transporter, putative	3.80	7.07E-06	0.007	t, m
LOC_Os03g15920	expressed protein	3.79	7.19E-05	0.047	
LOC_Os03g47610	thiamine biosynthesis protein thiC, putative	3.76	4.99E-06	0.005	pl
LOC_Os04g52250	LTPL123—Protease inhibitor/seed storage/LTP family protein precursor	3.73	6.36E-07	0.001	t
LOC_Os05g33840	transketolase, putative	3.64	1.57E-06	0.002	pl
LOC_Os01g40860	aldehyde dehydrogenase, putative	3.62	4.80E-05	0.033	
LOC_Os01g01340	light-induced protein 1-like, putative	3.48	2.20E-08	0.000	pl
LOC_Os08g35860	cytokinin dehydrogenase precursor, putative	3.47	4.90E-08	0.000	
LOC_Os04g16450	aquaporin protein, putative	3.47	6.90E-06	0.007	t, m
LOC_Os02g50960	auxin efflux carrier component, putative	3.10	1.34E-04	0.071	t, m
LOC_Os02g41630	phenylalanine ammonia-lyase, putative	3.02	3.51E-05	0.026	
LOC_Os12g29220	nodulin MtN3 family protein, putative	2.81	1.85E-05	0.016	t, m
LOC_Os08g04450	DAG protein, chloroplast precursor, putative	2.76	3.80E-05	0.028	pl
LOC_Os08g10080	no apical meristem protein, putative	2.64	3.08E-07	0.001	
LOC_Os05g31140	glycosyl hydrolases family 17, putative	2.60	2.21E-08	0.000	m
LOC_Os06g22960	aquaporin protein, putative	2.57	3.76E-07	0.001	t, m, pl
LOC_Os05g35470	dienelactone hydrolase family protein	2.56	1.93E-05	0.016	pl
LOC_Os03g50310	CCT/B-box zinc finger protein, putative	2.54	7.01E-05	0.046	m, pl
LOC_Os08g03310	zinc finger family protein, putative	2.35	1.34E-05	0.012	
LOC_Os03g20700	magnesium-chelatase, putative	2.28	1.07E-04	0.063	p, m, pl
LOC_Os09g35940	cytochrome P450, putative	1.76	2.01E-04	0.098	
LOC_Os09g29200	glutathione S-transferase, putative	-1.89	1.92E-04	0.096	c
LOC_Os03g20370	OsCam1-1—Calmodulin	-1.96	9.23E-05	0.057	c
LOC_Os03g28940	ZIM domain-containing protein, putative	-1.96	1.17E-04	0.068	
LOC_Os03g55240	cytochrome P450, putative	-1.97	2.03E-04	0.098	
LOC_Os11g47809	metallothionein, putative	-2.01	1.60E-05	0.014	
LOC_Os01g27210	glutathione S-transferase, putative	-2.09	8.78E-05	0.055	c
LOC_Os07g09340	plasma membrane ATPase, putative	-2.14	9.63E-05	0.058	
LOC_Os08g01160	Membrane-associated DUF588 domain-containing protein, putative	-2.18	1.67E-04	0.086	
LOC_Os10g38489	glutathione S-transferase GSTU6, putative	-2.23	1.65E-04	0.086	c
LOC_Os07g39350	transporter family protein, putative	-2.34	2.23E-06	0.003	
LOC_Os06g04070	pyridoxal-dependent decarboxylase protein, putative	-2.37	7.85E-05	0.050	
LOC_Os05g38880	expressed protein	-2.38	9.85E-05	0.059	
LOC_Os02g42990	OsSAUR11—Auxin-responsive SAUR gene family member	-2.56	1.34E-04	0.071	
LOC_Os06g48250	ATPase, putative	-2.66	1.87E-04	0.094	
LOC_Os01g41010	DUF581 domain containing protein	-2.66	1.87E-05	0.016	
LOC_Os12g04500	response regulator receiver domain-containing protein	-2.66	4.87E-05	0.033	
LOC_Os10g31330	retrotransposon protein, putative, unclassified	-2.69	4.08E-06	0.004	
LOC_Os11g04720	OsRR10 type-A response regulator	-2.72	3.98E-06	0.004	
LOC_Os03g53540	expressed protein	-2.73	1.27E-04	0.070	
LOC_Os04g37490	oxidoreductase, aldo/keto reductase family protein, putative	-2.80	1.48E-05	0.013	
LOC_Os01g04330	OsCML16—Calmodulin-related calcium sensor protein	-2.95	1.64E-04	0.086	
LOC_Os07g12890	metal cation transporter, putative	-3.00	6.73E-06	0.007	
LOC_Os02g20360	tyrosine aminotransferase, putative	-3.13	9.18E-05	0.057	
LOC_Os03g11900	transporter family protein, putative	-3.13	2.62E-13	0.000	
LOC_Os07g32060	UDP-glucoronosyl/UDP-glucosyl transferase, putative	-3.14	1.01E-04	0.060	
LOC_Os12g39360	aspartic proteinase nepenthesin precursor, putative	-3.20	2.12E-07	0.000	
LOC_Os05g50100	expressed protein	-3.25	1.47E-05	0.013	
LOC_Os03g10100	transporter family protein, putative	-3.34	2.33E-05	0.019	
LOC_Os01g72530	OsCML31—Calmodulin-related calcium sensor protein	-3.38	1.28E-04	0.070	c
LOC_Os01g45110	anthocyanin 3-O-beta-glucosyltransferase, putative	-3.76	3.49E-06	0.004	
LOC_Os01g28450	SCP-like extracellular protein	-3.84	3.45E-06	0.004	
LOC_Os05g38860	expressed protein	-4.05	2.14E-05	0.017	
LOC_Os07g48800	VQ domain-containing protein, putative	-4.30	2.04E-04	0.098	
LOC_Os06g05420	expressed protein	-4.43	1.29E-04	0.070	
LOC_Os04g47360	OsPOP9—Putative Prolyl Oligopeptidase homolog	-4.46	9.85E-08	0.000	
LOC_Os06g48560	transferase family protein, putative	-4.81	6.09E-06	0.006	
LOC_Os03g58300	indole-3-glycerol phosphate lyase, chloroplast precursor, putative	-6.49	4.30E-05	0.030	
LOC_Os03g60580	actin-depolymerizing factor, putative	-6.57	7.06E-08	0.000	
LOC_Os03g58320	indole-3-glycerol phosphate lyase, chloroplast precursor, putative	-6.59	4.58E-05	0.032	
LOC_Os04g43200	caleosin-related protein, putative	-6.71	1.18E-04	0.068	
LOC_Os09g34230	UDP-glucoronosyl/UDP-glucosyl transferase, putative	-6.87	2.47E-08	0.000	
LOC_Os11g03760	expressed protein	-6.90	2.39E-07	0.001	
LOC_Os06g46740	early nodulin 20 precursor, putative	-8.65	1.70E-04	0.086	
LOC_Os07g45080	expressed protein	-9.36	3.56E-06	0.004	
LOC_Os01g10110	cytokinin dehydrogenase precursor, putative	-9.52	4.81E-07	0.001	
LOC_Os03g17790	OsRCI2-5—Putative low-temperature and salt-responsive protein	-10.43	2.10E-08	0.000	
LOC_Os10g18150	crooked neck, putative	-15.84	8.75E-07	0.001	
LOC_Os05g46480	late embryogenesis abundant protein, group 3, putative	-17.33	6.11E-05	0.041	
LOC_Os11g26790	dehydrin, putative	-26.49	8.16E-05	0.052	c
LOC_Os01g72090	*SE13/OsHY2*, phycoerythrobilin ferredoxin oxidoreductase, putative	-35.33	1.23E-04	0.069	f
LOC_Os01g72120	glutathione S-transferase, putative	-35.97	1.23E-04	0.069	c
LOC_Os01g72130	glutathione S-transferase, putative	-84.91	1.65E-14	0.000	c
LOC_Os01g72100	OsCML10—Calmodulin-related calcium sensor protein	-115.59	7.53E-18	0.000	c
LOC_Os01g72170	glutathione S-transferase, putative	-259.89	1.27E-36	0.000	c

According to Gene Ontology (GO) terms, the classification of the differentially expressed genes in *G123* indicated that the mutant showed major alterations in photosynthesis, and in other processes related to the response to light. The functional annotation, as well as the assignment of the functional categories to the 116 genes based on their GO, were carried out with the Comprehensive Annotation of Rice Multi-Omics database (CARMO, http://bioinfo.sibs.ac.cn/carmo). The biological process classification according to the GO term annotation of the 62 up-regulated genes in *G123* revealed that the bulk of these genes were included in the groups related to transport, photosynthesis, light harvesting, and responses to blue, red and far red light (Table 3). Of the genes related to the response to light, at least eight genes also affected the chlorophyll-binding proteins. The cellular component classification clearly showed that all the GO terms were directly associated with chloroplast or membranes. Thus, the terms related to the organelle were very abundant, e.g. those defined as thylakoid, thylakoid membrane, chloroplast envelope, plastoglobuli, chloroplast stroma or the light-harvesting complex. In particular, the biological function classification grouped the genes mostly included in the GO terms involving general binding, metal ion binding or chlorophyll binding.

**Table 3:** GO classification of the up-regulated genes in *G123*.

GO Term	Description	Count	%	p-value	FDR
***Biological process***					
GO:0006810	transport	14	23.0	7.31E-02	4.06E-03
GO:0015979	photosynthesis	11	18.0	2.08E-08	1.04E-08
GO:0009765	photosynthesis, light harvesting	7	11.5	3.52E-10	3.52E-10
GO:0009637	response to blue light	5	8.2	3.80E-04	4.22E-05
GO:0006091	generation of precursor metabolites and energy	5	8.2	2.87E-01	1.10E-02
GO:0010218	response to far red light	4	6.6	1.11E-02	8.56E-04
GO:0010114	response to red light	4	6.6	1.31E-02	9.37E-04
GO:0019344	cysteine biosynthetic process	4	6.6	3.50E-02	2.18E-03
GO:0009228	thiamine biosynthetic process	3	4.9	2.52E-02	1.68E-03
GO:0010154	fruit development	3	4.9	9.58E-02	4.56E-03
GO:0010155	regulation of proton transport	3	4.9	1.52E-01	6.90E-03
***Cellular Component***					
GO:0016020	membrane	23	37.7	7.82E-02	4.12E-03
GO:0009536	plastid	23	37.7	8.33E-02	4.17E-03
GO:0009507	chloroplast	15	24.6	2.81E-04	3.51E-05
GO:0005622	intracellular	11	18.0	2.12E-01	8.48E-03
GO:0009579	thylakoid	9	14.8	1.34E-03	1.34E-04
GO:0009535	chloroplast thylakoid membrane	8	13.1	1.17E-04	1.67E-05
GO:0009941	chloroplast envelope	8	13.1	2.02E-03	1.84E-04
GO:0010287	plastoglobule	6	9.8	1.10E-05	1.83E-06
GO:0009570	chloroplast stroma	6	9.8	1.75E-01	7.62E-03
GO:0030076	light-harvesting complex	5	8.2	1.21E-06	3.03E-07
GO:0009534	chloroplast thylakoid	5	8.2	5.15E-03	4.30E-04
***Molecular Function***					
GO:0046872	metal ion binding	19	31.1	8.46E-06	1.69E-06
GO:0005488	binding	19	31.1	5.15E-02	3.03E-03
GO:0005215	transporter activity	10	16.4	2.09E-01	8.48E-03
GO:0016168	chlorophyll binding	6	9.8	6.49E-08	2.16E-08

Thresholds: p-value < 0.5; FDR < 0.1

Regarding the down-regulated genes in *G123*, the biological process classification revealed that most numerous groups of genes corresponded to the GO terms related to metabolic processes and response to internal stimuli, which included 21 and 10 genes, respectively (Table 4). Similarly, the most relevant group of genes in the cellular component classification was included in the term cytoplasm, while the molecular function classification grouped the genes associated with transferase and transporter activity.

**Table 4:** GO classification of the down-regulated genes in *G123*.

GO Term	Description	Count	%	p-value	FDR
***Biological process***					
GO:0008152	metabolic process	21	42.0	4.54E-01	4.13E-02
GO:0009719	response to endogenous stimulus	10	20.0	2.02E-02	6.73E-03
GO:0009056	catabolic process	9	18.0	8.10E-02	1.35E-02
GO:0019748	secondary metabolic process	6	12.0	5.24E-02	1.31E-02
***Cellular Component***					
GO:0005737	cytoplasm	10	20.0	7.86E-02	1.35E-02
***Molecular Function***					
GO:0016740	transferase activity	11	22.0	1.52E-01	2.17E-02
GO:0005509	calcium ion binding	5	10.0	3.30E-01	3.67E-02
GO:0022891	substrate-specific transmembrane transporter activity	3	6.0	2.36E-01	2.95E-02
GO:0022857	transmembrane transporter activity	3	6.0	4.44E-01	4.13E-02

Thresholds: p-value < 0.5; FDR < 0.1

It is worth mentioning that the changes in the expression of genes involved in *G123* flowering regulation were also observed, as expected, in an early flowering mutant. In *G123*, *Hd3a* (LOC_Os06g06320), one of the master genes to induce flowering was up-regulated by 37.9-fold. Similarly, other genes implicated in flowering, such as *MADS14* (LOC_Os03g54160) and *MADS18* (LOC_Os07g41370), which act downstream of *Hd3a*, also displayed expression levels that were 20.5- and 3.8-fold higher in *G123* (Table 2).

### Detection of the mutation

Several approaches were adopted to identify the putative gene responsible for the early flowering phenotype of *G123*. First, the differentially expressed genes identified in the transcriptome analysis were represented on a volcano plot, on which statistical significance, given by the p-value in–log_10_, was plotted against the variation of expression given by the log_2_ of fold change (S1 Fig). Three of the most down-regulated genes, LOC_Os01g72170, LOC_Os01g72130 and LOC_Os01g72120, encoded the proteins with the glutathione S-transferase function. A fourth gene, LOC_Os01g72100, encoded a calmodulin-like protein. The fifth most down-regulated gene compared to the wild type, LOC_Os01g72090, encoded *SE13/OsHY2*, a phytochromobilin synthase involved in both the biosynthesis of phytochromes and the response of plants to the photoperiod. Interestingly, these five genes are located in a 29796 bp region in chromosome 1, between positions 41825087 and 41854883. This observation suggests that the down-regulation of these five genes may be due to a deletion in that region.

In order to investigate the mutations produced by the irradiation that affects the heading date in *G123*, we generated an F_2_ population that derived from a cross between Gleva and *G123*. The nuclear DNA from the leaf samples of Gleva, *G123* and from a bulk (Epool) of the 20 F_2_ plants showing early flowering, similar to the phenotype exhibited by *G123*, was sequenced and compared.

In an attempt to detect variations among the genomes of Epool, *G123* and the wild type, an analysis of structural variations (SV) was done with a combination of two programs: LUMPY and SVTYPER [27, 28]. The SV search included mutations larger than 50 pb, which comprised deletions, duplications, insertions, inversions, and intra- and inter-chromosomal translocations. Different SV types in the genome sequences of the three DNA samples were detected by making a comparison with the reference genome and those found in both *G123* and the Epool, but not in Gleva (see Materials and Methods), and were selected for further analyses. Ten SVs, consisting in five deletions, four translocations (each represented by two entries in the table) and one inversion, remained after filtering (Table 5). After hand curation in the IGV browser software, only the occurrence of one variation was fully confirmed: a deletion of 33373 bp located in chromosome 1 at position 41822688–41856061 pb. Eight genes were present in this region and are indicated in Table 6. Most of these genes were previously identified in the RNA-seq analysis (Table 2) as the genes that exhibited the highest differential expressions between *G123* and Gleva. Six of them encoded proteins with glutathione S-transferase activity, and one of the remaining two genes was CML10, a calmodulin-like protein. Interestingly, the other gene encoded *SE13/OsHY2*, a gene involved in the response of plants to light and is, therefore, putatively implicated in the early flowering phenotype observed in the mutant line *G123*. This also agrees with the lack of *SE13* expression observed in the *G123* plants (Fig 3).

**Table 5:** Structural variations specific of *G123* and Epool that were absent in Gleva.

Chromosome	Position	Variation type
**Chr1**	41822688	deletion
**Chr2**	23819730	deletion
**Chr5**	29186488	translocation
**Chr8**	2878658	translocation
**Chr8**	5037462	translocation
**Chr8**	8430256	inversion
**Chr9**	21044854	translocation
**Chr10**	15910040	deletion
**Chr10**	16389174	deletion
**Chr10**	3604760	translocation
**Chr10**	15846229	translocation
**Chr11**	13243899	translocation
**Chr12**	10873676	deletion
**Chr12**	10056201	translocation

**Table 6:** Functional genes located in the deleted region in the *G123* genome. Functional genes located between positions Chr1:414822688 and Chr1:41856061, corresponding to the deleted region in the *G123* genome.

MSU	Gene Symbol	Gene Name
LOC_Os01g72090	*SE13*	*Photosensitivity 13*
LOC_Os01g72100	*CML10*	*Calmodulin-like protein 10*
LOC_Os01g72120	*GSTU7*	*Tau glutathione S-transferase 7*
LOC_Os01g72130	*GSTU35*	*Tau glutathione S-transferase 35*
LOC_Os01g72140	*GSTU36*	*Tau glutathione S-transferase 36*
LOC_Os01g72150	*GSTU37*	*Tau glutathione S-transferase 37*
LOC_Os01g72160	*GSTU41*	*Tau glutathione S-transferase 41*
LOC_Os01g72170	*GSTU42*	*Tau glutathione S-transferase 42*

## Discussion

The rice mutant line *G123* was identified in a screening for early flowering plants. In addition to early flowering, the *G123* mutant also exhibited photoperiod insensitivity, which suggests that its mutation affects photoperiod-mediated flowering regulation. The analysis of the structural variations in the *G123* genome indicates that *SE13/OsHY2* is the most probable candidate responsible for the early flowering phenotype. *SE13/OsHY2* encodes a phytochromobilin synthetase that participates in the last step of the synthesis of phytochromobilin, a chromophore that forms part of the phytochrome structure [19]. Phytochromes participate in photoperiod flowering regulation as they inhibit *Hd3a* under LD conditions through *Hd1*, and also repress *RFT1* expression by inhibiting *Ehd1* [31]. Consequently, plants defective in phytochrome due to lack of *SE13/OsHY2* functionality should flower earlier than those plants with functional phytochromes, as observed in *G123*.

The *SE13/OsHY2* gene was first described in *X61*, a Gimbozu mutant [21], in which a deletion of a single nucleotide in the first exon caused a shift in the reading frame to produce a premature stop codon. Consequently, the mutant line flowered 35 days earlier than Gimbozou under natural LD light conditions, similarly to *G123*. When comparing the expression patterns of the main flowering regulatory genes in *X61* and *G123* under the LD conditions, as expected *Hd3a* displayed higher levels in both mutant lines compared to their corresponding wild types. The *RFT1* levels were higher in Gleva than in *G123*, probably due to their differences in vegetative cycle duration. Despite there being some connections between the two flowering regulatory pathways, *Hd3a* expression is regulated by *Hd1* and *Ehd1*, while *RFT1* expression is regulated by *Ehd1* [32]. In regions located at northern latitudes with a temperate climate, varieties can often be found with non-functional *Hd1* alleles. As *Hd1* is an inhibitor of flowering under LD conditions, in these situations, flowering is governed by *Ehd1*. This is not the case of Gleva because it contains an *Hd1* functional allele, which implies that both regulatory pathways are functional in Gleva [6]. This agrees with the fact that the *Hd3a* expression levels in the *G123* mutant are higher than in the wild type, which indicates that *Hd3a* also promotes flowering in *G123*.

In previous studies by our research group, another mutant that exhibits photoperiod insensitivity, *s73*, was isolated in an irradiated Bahia variety collection. The identification of a null mutation in *SE5*, and the analysis of *Ehd1* silencing in both Bahia and *s73* backgrounds, not only proved that *SE5* regulates *Ehd1* expression, but *SE5* also confers photoperiodic sensitivity through *Hd1* regulation. These results provided direct evidence that phytochromes inhibit flowering by negatively modulating both the *Hd1* and *Ehd1* flowering pathways [20]. *SE5* encodes a hemoxygenase that acts one step upstream of *SE13/OsHY2* in the phytochromobilin synthesis pathway to produce the substrate of *SE13/OsHY2*, a molecular connection that explains why *s73* plants present similar alterations to *G123*. The *Hd3a* expression in *s73* displayed much higher levels than those in the non-mutated parental variety, as in *G123* and *X61*. Moreover, the expression of *Ehd1*, an *Hd3a* inductor, also peaked and was much higher than that observed in the non-mutated line at 4 h after dawn under LD conditions, which agrees with lack of *Ehd1* inhibition by phytochromes. This reinforces the hypothesis that *Hd3a* also induces flowering in the *G123* mutant.

The early flowering phenotype of *G123* was also reflected in the alteration of the other genes participating in the flowering regulation pathway. *HD5/DTH8/Ghd8* codes for a HEME ACTIVATOR PROTEIN 3 (HAP3), a subunit of the CCAAT-box-binding transcription factor complex [33]. It acts as a repressor of flowering under LD conditions, and delays flowering by inhibiting the expression of *Ehd1* and, consequently, of *Hd3a* and *RFT1* [34]. Conversely under SD conditions, *Ghd8* induces the expression of these regulators [33]. *Ghd8* expression is not affected by *Ghd7* or *Hd1*, which indicates the occurrence of a different genetic pathway in the control of flowering [34]. The *Ghd8* and *Ghd7* expression patterns in the *G123* mutant were similar under LD conditions and their levels were lower than in Gleva, which is in agreement with the observed early flowering in the mutant. As the expression of both genes is activated by light [8], regulation by phytochromes is altered in *G123*. It is noteworthy that in the mutant, both *Ghd7* and *Ghd8* presented peaked induction at 12 h after dawn in the dark phase under SD conditions and may, thus, act as inductors of flowering in the SD photoperiod (Fig 3).

*ELF3-1* promotes rice flowering under the LD conditions by inhibiting *Ghd7* expression [35]. *ELF3-1-*defective plants exhibit higher levels for the *Ehd1*, *RFT1* and *Hd3a* expressions under LD [36]. *OsELF3-1* might be involved in PhyB-mediated flowering regulation as it has been reported that *oself3-1* mutation suppresses the photoperiod-insensitive early flowering of *se5* [37]. Furthermore, *Ghd7* expression is activated by pulses of light at higher rates in *ef7*, a mutant defective in *ELF3-1*, than in wild-type plants [21]. In Arabidopsis, it has been demonstrated that ELF3 interacts directly with PhyB and other proteins to form complexes capable of regulating the gene expression of several flowering regulatory pathway genes [38]. Recently, an interaction between OsELF3 and PhyB has been demonstrated in yeast cells [37]. We have previously shown that Gleva exhibits higher *OsELF3-1* levels and lower *Ghd7* levels than *G123* under LD. Under these conditions, the accumulation of phytochromes is greater due to the number of light hours in accordance with lack of phytochrome action in *G123*.

*Hd6* encodes an α-subunit of protein kinase CK2 (CK2α) and requires a functional *Hd1* gene to perform its function by acting independently of circadian clock mechanisms [39, 40]. In our analysis, we observed that Gleva displayed higher *Hd6* expression levels when *Hd1*expression peaked, which occurred at the end of the dark period under both SD and LD conditions. This observation agrees with the flowering times of both Gleva and the mutant. Finally, *Days to heading on chromosome 2* (*DTH2*) encodes an Hd1-like protein that induces *Hd3a* and *RFT1* expressions by acting independently of *Hd1* and *Ehd1*. The circadian clock regulates *DTH2* expression [41], a gene that increased in the dark phase of the day under SD and LD conditions in both *G123* and Gleva, and with lower *G123* levels than in Gleva.

The phenotypic data and the transcriptome analysis of the *G123* mutant indicated that the deletion detected in *SE13/OsHY2* was very likely responsible for the altered phenotype of *G123*. Consistently with the defect in phytochrome content, the transcriptome analysis revealed that photosynthesis and other processes related to the response to light were profusely altered in *G123*. A major group of genes corresponding to transport and photosynthesis was up-regulated in not only the *G123* plants, but also in other genes involved in the response to light, in relation to different chloroplast elements, such as thylakoids or stroma. Therefore, the role of *SE13/OsHY2* in the synthesis of phytochromes and its function in flowering regulation could explain the phenotype observed in the mutant.

In the last few years, several methodologies based on whole genome sequencing have been developed to detect the mutations responsible for altered phenotypes. In our case, in order to identify the mutation responsible for the early flowering phenotype exhibited by *G123*, we used a structural variation detection pipeline that combines two programs, LUMPY and SVTYPER [27, 28], which were initially developed to detect such variations in human genomes. Using these tools in conjunction with a pooled F_2_ generation, we avoided employing several generations of plants given the time that this entails. It is worth mentioning that our attempts to use another method to identify the *G123* mutation, such as MutMap, developed to identify single nucleotide polymorphisms (SNP) mutations [42], were not successful given its restriction to detect SNP-type mutations. However, the methodology used herein allowed the detection of a deletion of 29.8 Kb, which is most probably responsible for the observed phenotype. The combination of SV detection and a pooled F_2_ is a novel methodology for detecting mutations in plants. It generates only a few false-positives, enables easy hand curation, and offers the possibility of reducing the time spent to identify mutations.

## Conclusions

This manuscript reports the generation and identification of a mutant, *G123*, that displays an early flowering phenotype. The proposed structural variation responsible for the mutation was identified by an analysis technique that combines LUMPY and SVTYPER [27, 28] in conjunction with a pooled F_2_ population. This approach suggests that *SE13/OsHY2*, a gene encoding a phytochromobilin synthase implicated in phytochrome chromophore biosynthesis, is the candidate gene for the altered phenotype of the mutant. The expression analysis of the major flowering regulatory genes indicated that, in the absence of functional phytochromes, flowering in the *G123* mutant was governed mainly by *Hd3a* rather than by *RFT1* under LD conditions. We also revealed that the *G123* transcriptome reveals major alterations in the expression of a group genes involved in both photosynthesis and the light response. The *SE13/OsHY2* gene is proposed as an interesting donor in breeding programs to reduce the vegetative cycle of elite varieties.

## Supporting information

S1 Fig Volcano plot analysis of the differential expressed genes in *G123*. Statistical significance, given by the–log_10_ of the p-value (ordinate axis), is plotted against the variation of expression given by the log_2_ of fold change (abscissa axis) for each gene.
(TIF)

S1 Table Sequences of primers and temperature (°C) used in the quantitative RT-PCR analysis. (DOCX)

S2 Table Statistics of SNP detection and annotation in Gleva, *G123* and Epool. Nipponbare MSU v7 was used as a reference.
(XLSX)

S3 Table Statistics of mapping rate, depth and coverage. (XLSX)

S4 Table Data from the statistical analysis of SV detection and annotation in the Gleva, *G123* and Epool DNA samples. (XLSX)
