# Supplementary figures and images for: The G123 rice mutant, carrying a mutation in SE13, presents alterations in the expression patterns of photosynthetic and major flowering regulatory genes
Source: PLoS One. 2020 May 18;15(5):e0233120. doi: 10.1371/journal.pone.0233120 (PMC7233571; doi:10.1371/journal.pone.0233120)

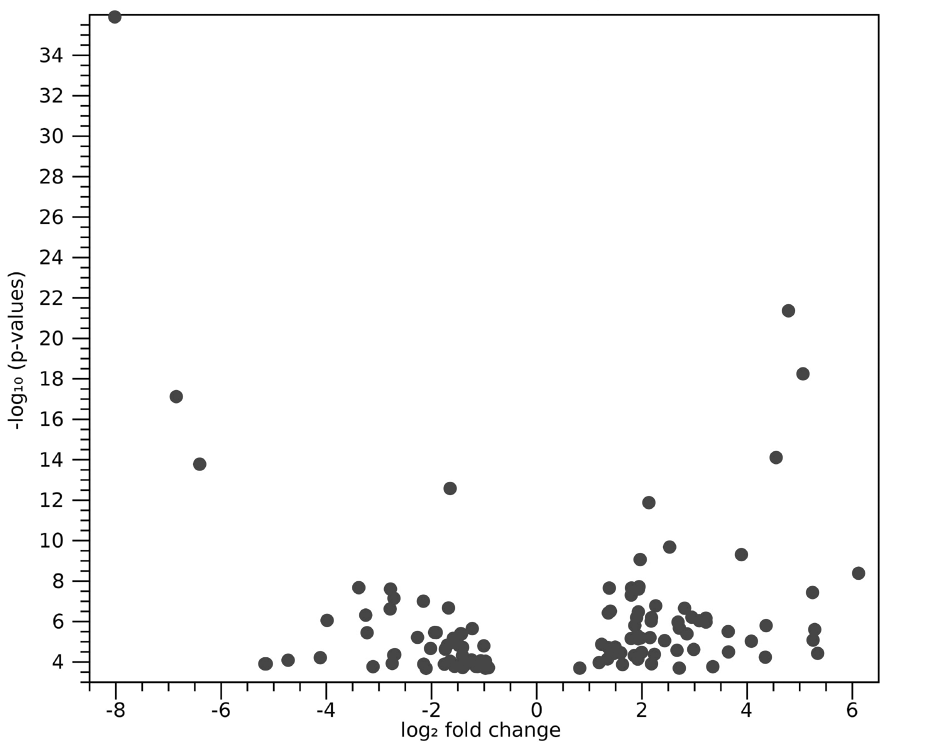

Supplement: S1 Fig — Statistical significance, given by the–log10 of the p-value (ordinate axis), is plotted against the variation of expression given by the log2 of fold change (abscissa axis) for each gene. (TIF) [file pone.0233120.s001.tif]
