# Supplementary material for: The G123 rice mutant, carrying a mutation in SE13, presents alterations in the expression patterns of photosynthetic and major flowering regulatory genes
Source: PLoS One. 2020 May 18;15(5):e0233120. doi: 10.1371/journal.pone.0233120 (PMC7233571; doi:10.1371/journal.pone.0233120)
Supplement: S1 Table — (DOCX) [file pone.0233120.s002.docx]

**S1 Table.- Sequences of primers, temperature (°C) and number of cycles used in quantitative RT-PCR analysis**.

| Gene | Locus | Primer Sequence F/R (5’-3’) | T (^o^C) | cycles |
| --- | --- | --- | --- | --- |
| *DTH2* | LOC_Os02g49230 | GATTTCTGCAGGGAGCAAAG / TTCAAGACAACGGACTGCTG | 60 | 45 |
| *Ehd1* | LOC_Os10g32600 | TCGGAGAAGACAAGGCAGTT /CCGTGTTTGCTTGTTGTTGG | 59 | 45 |
| *ELF3* | LOC_Os06g05060 | ACTACTTCCCGCCTTTCAGC /ATCCACGACTGCTGCTCAAA | 60 | 45 |
| *Ghd7* | LOC_Os07g15770 | TATTGTGGGAGCACGTTCAC / ATCTGAACCATTGTCCAAGC | 57 | 45 |
| *Ghd8* | LOC_Os08g07740 | CGAAGGAGCAGGACAGGTTC / AGCTGATGAACTCCGACACG | 62 | 45 |
| *Hd1* | LOC_Os06g16370 | CTTACACAGATTCCATCAGC / CATACGCCTTTCTTGTTTCA | 55 | 45 |
| *Hd3a* | LOC_Os06g06320 | GATGCACCAAGCCCAAGT / GGAACAGCACGAACACCA | 61 | 45 |
| *HD6* | LOC_Os03g55389 | GTTCAATGGGGTGAGCAGGA / CTTCACAGGCTTGAGTATCTTGA | 59 | 45 |
| *OsGI* | LOC_Os01g08700 | GTGCCGTCTATCAACCACCA / AAGGACGGACATGCTGAGTG | 60 | 45 |
| *PRR37* | LOC_Os07g49460 | CCTATGGCAGCATGTGTGGA / ACCATCGTCGTCATCATCGT | 60 | 45 |
| *RFT1* | LOC_Os06g06300 | GGATTGAACGGCAGGAGATA /CGGCCATGTCAAATTAATAACC | 60 | 45 |
| *Ubiquitin* | LOC_Os03g13170 | GCTCCGTGGCGGTATCAT / CGGCAGTTGACAGCCCTAG | 55 | 45 |
